# Eddy covariance and biometric measurement**s** show that a savanna ecosystem in Southwest China is a carbon sink

**DOI:** 10.1038/srep41025

**Published:** 2017-02-01

**Authors:** Xuehai Fei, Yanqiang Jin, Yiping Zhang, Liqing Sha, Yuntong Liu, Qinghai Song, Wenjun Zhou, Naishen Liang, Guirui Yu, Leiming Zhang, Ruiwu Zhou, Jing Li, Shubin Zhang, Peiguang Li

**Affiliations:** 1 Key Laboratory of Tropical Forest Ecology, Xishuangbanna Tropical Botanical Garden, Chinese Academy of Sciences, Mengla, Yunnan 666303, China; 2 University of Chinese Academy of Sciences, Beijing 100049, China; 3 Yuanjiang Savanna Ecosystem Research Station, Xishuangbanna Tropical Botanical Garden, Chinese Academy of Sciences, Yuanjiang, Yunnan 653300, China; 4 Global Carbon Cycle Research Section, Center for Global Environmental Research, National Institute for Environmental Studies, Tsukuba, 305-8506, Japan; 5 Synthesis Research Center of Chinese Ecosystem Research Network, Key Laboratory of Ecosystem Network Observation and Modeling, Institute of Geographic Sciences and Natural Resources Research, Chinese Academy of Sciences, Beijing 100101, China

## Abstract

Savanna ecosystems play a crucial role in the global carbon cycle. However, there is a gap in our understanding of carbon fluxes in the savanna ecosystems of Southeast Asia. In this study, the eddy covariance technique (EC) and the biometric-based method (BM) were used to determine carbon exchange in a savanna ecosystem in Southwest China. The BM-based net ecosystem production (NEP) was 0.96 tC ha^−1^ yr^−1^. The EC-based estimates of the average annual gross primary productivity (GPP), ecosystem respiration (R_eco_), and net ecosystem carbon exchange (NEE) were 6.84, 5.54, and −1.30 tC ha^−1^ yr^−1^, respectively, from May 2013 to December 2015, indicating that this savanna ecosystem acted as an appreciable carbon sink. The ecosystem was more efficient during the wet season than the dry season, so that it represented a small carbon sink of 0.16 tC ha^−1^ yr^−1^ in the dry season and a considerable carbon sink of 1.14 tC ha^−1^ yr^−1^ in the wet season. However, it is noteworthy that the carbon sink capacity may decline in the future under rising temperatures and decreasing rainfall. Consequently, further studies should assess how environmental factors and climate change will influence carbon-water fluxes.

Savanna ecosystems are characterized by distinct wet and dry seasons, the codominance of C3 trees and C4 grasses^1^, and their location mainly in the tropics and subtropics. They cover almost 60%, 50%, and 45% of the areas of Africa, Australia, and South America, respectively, and more than 10% of the area of Southeast Asia^2^. They play an increasing role in the carbon cycle and energy fluxes in the context of climate changes (e.g., decreasing precipitation and increasing temperature), as they cover approximately 20.0% (2.7 billion ha) of the global land surface^1^,^3^,^4^,^5^ and account for ~30% of the net primary production (NPP) of the terrestrial ecosystem^6^. Savannas also have a large and rapidly growing human population: about one-fifth of the global population is supported by savanna ecosystems^3^,^4^,^7^. The carbon exchange of savanna ecosystems, therefore, has a significant influence on global carbon cycling. Consequently, research on the spatiotemporal characteristics of carbon exchange and its responses to biotic and abiotic controls on savanna ecosystems is of great importance, not only for improved fundamental ecological understanding of the impact of global change on carbon fluxes but also for the improved protection and management of this vulnerable ecosystem type for sustainable development and provision of better ecosystem services (e.g., the management of resources, water, biodiversity, and climate change).

There have been many studies of carbon exchange and its variations in savanna ecosystems in Africa^8^,^9^,^10^,^11^,^12^,^13^,^14^, Australia^15^,^16^,^17^,^18^,^19^, and South America^20^,^21^. Carbon exchange between savanna ecosystems and the atmosphere varies both seasonally and interannually even in the same site, let alone among different sites. According to published studies, most savanna ecosystems are appreciable carbon sinks^10^,^12^,^19^,^21^,^22^,^23^. Some are carbon neutral or marginal carbon sources^9^,^13^,^24^ and show considerable seasonal fluctuations^8^,^9^,^10^,^13^,^25^,^26^,^27^,^28^ due to variations and uncertainties in rainfall, water availability, solar radiation, temperature, terrain, nutrients, fire, and human activity^4^,^11^,^29^,^30^,^31^,^32^, while others are carbon sources^13^,^33^,^34^. However, there has been little research on carbon flux and its variation in Southeast Asia; to our knowledge, only one related study has been conducted in a tropical savanna in India to investigate the impact of rainfall and grazing on NPP using biometrics^35^. So far, there have not been any related studies on carbon exchange in savanna ecosystems in China, because typically such savannas are located in hot-dry valleys surrounded by winding mountains, where the difficulty of access makes plot arrangement, observations, and instrument maintenance difficult. Savanna ecosystems play an important role in mitigating global warming^5^ and would likely be more sensitive to global changes than forest ecosystems^8^,^36^,^37^,^38^. As there has not been any research on how and why carbon fluxes change, particularly seasonally, over savanna ecosystems in China, it is necessary to conduct studies to answer these questions and to explore the differences between carbon exchange from savannas in China, Africa, Australia, South America, and other savanna ecosystems. We start by looking at the distribution of savanna ecosystems in China.

Savannas in China are distributed mainly in the basins or valleys in Yunnan, Guizhou and Sichuan provinces, the northwestern part of the island of Hainan, southern Taiwan, and the coastal hills of Guangdong. The total area of savannas in Southwest China is ~8 × 10^6^ ha^39^. The Yuanjiang savanna, where our study is carried out, is the most typical and representative of Chinese savannas^40^,^41^. In addition, the savanna here is similar to Indian and African savannas in terms of its vegetation and the structure and species of the flora community^41^. Yuanjiang savanna is characterized by a hot-dry climate because of the large amounts of solar and net radiation it receives^42^, its low rainfall, and the Foehn effect^40^. Prior to our study, we had no idea of the net ecosystem carbon exchange (NEE) of this typical savanna ecosystem and its variations, whether diurnal or seasonal. Furthermore, understanding the state of carbon sequestration and seasonal fluctuation is beneficial, not only for understanding the important role of savannas in the global carbon cycles and predicting future carbon exchanges under climate change^5^, but also for developing policies or management practices to protect similar ecosystems that would likely be more sensitive to climate change.

In this study, a biometric-based method (BM) and an eddy-covariance (EC) method were applied to investigate carbon exchange. We used the BM to measure the net ecosystem production (NEP) and EC to measure the net ecosystem carbon exchange (NEE) of the Yuanjiang savanna ecosystem in Southwest China during the period May 2013 to December 2015. The main objectives of this study are: 1) to quantify the gross primary productivity (GPP), ecosystem respiration (R_eco_), and NEE to determine whether this region is a carbon sink or source; 2) to understand the diurnal and seasonal variation in carbon fluxes; and 3) to explore carbon uptake responses to climate change.

## Results

### Wind rose and footprint

**Figure 1:**
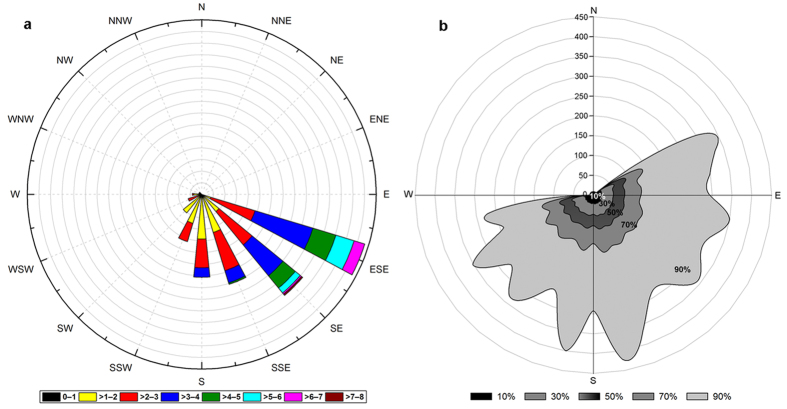
Wind rose and footprint of the eddy covariance system in the savanna ecosystem of the study site in Southwest China. (**a**) The direction of the stripe shows the wind direction (°) and the color of the stripe indicates wind speed (m/s); (**b**) footprint (m).

The wind rose gives us the prevailing wind direction and speed, and the footprint gives the location of the EC system measurements. The wind rose for a whole year (2015) of observations (Fig. 1) shows that the prevailing wind directions at the study site are ESE and SE, and the wind speed mainly lies in the range 2–7 m s^−1^ (Fig. 1a), while the footprint shows that most (90%) of the carbon fluxes measured by the EC system are in an area within 500 m of the flux tower (Fig. 1b). In addition, the results confirm that the 1 ha permanent plot is within the footprint of our flux tower, which makes the comparison of NEP and NEE more meaningful and reliable.

### Ecosystem carbon budget

#### Biometric-based NEP and carbon use efficiency

The inventoried biomass, litterfall (for details on the seasonal and annual variations, see Supplementary Fig. S1), and measured R_h_ data gave values for ΔB, L_p_, and R_h_ of 1.97, 2.14, and 3.16 tC ha^−1^ yr^−1^ between 2014 and 2015, respectively, and the biometric-based NEP was estimated from equation (1) to be ~0.96 tC ha^−1^ yr^−1^ in 2015 (Table 1). Carbon use efficiency (CUE), which reflects the capacity of forests to absorb CO_2_ from the atmosphere and fix it in terrestrial biomass and the influence of autotrophic respiration on GPP in forests, is defined as the ratio of NPP to GPP, giving a value of 0.60 (Table 1).

#### Eddy covariance carbon exchange and its variations

**Figure 2:**
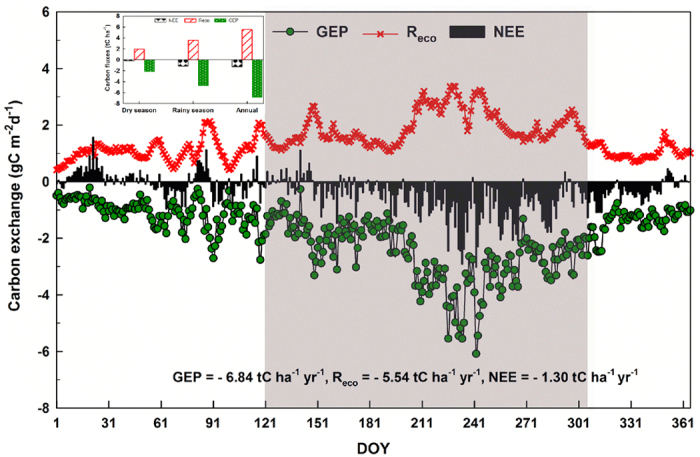
Averaged intra-annual variations in carbon exchange between May 2013 and December 2015 in the studied savanna ecosystem, Southwest China. GEP, R_eco_, and NEE indicate gross ecosystem productivity, ecosystem respiration, and net ecosystem carbon exchange, respectively. The amounts of GEP, R_eco_, and NEE for the dry season, wet season, and annually are shown in the inset at upper left. Shaded area indicates the wet season (May–October) and the rest of the figure area is the dry season (November–April).

Using 32 months (May 2013 to Dec 2015) of data, the amplitude of the averaged daily NEE ranged from −1.28 to 1.57 gC m^−2^ d^−1^ (the largest net carbon release was on 22 January) in the dry season, and −3.03 (maximum net carbon uptake was on 30 August) to 0.65 gC m^−2^ d^−1^ during the wet season (Fig. 2). The peak values of mean gross ecosystem productivity (GEP) and R_eco_ were both observed in August: GEP (GEP = −GPP) reached a peak of −6.09 gC m^−2^ d^−1^ on 30 August, and the maximum value of R_eco_ was 3.37 gC m^−2^ d^−1^ on 17 August. Strong seasonality in NEE (NEE = −NEP) was observed at the study site (Fig. 2). The site is almost carbon neutral (i.e., the total sum of CO_2_ absorbed by photosynthesis is nearly equal to that released by ecosystem respiration) in the dry season (−0.16 tC ha^−1^), but appreciable CO_2_ uptake was observed in the wet season (−1.14 tC ha^−1^) during the study period. A comparison of seasonal values of GEP and R_eco_ shows large variations between the dry season (−2.12 tC ha^−1^ and 1.96 tC ha^−1^, respectively) and the wet season (−4.72 tC ha^−1^ and 3.58 tC ha^−1^, respectively). Overall, the respiration rate of the ecosystem in the wet season is approximately 1.8 times that in the dry season, whereas the photosynthesis rate during the wet season is ~2.2 times that during the dry season, i.e., the wet season GEP was ~70% of the total annual GEP. Thus, the savanna ecosystem served as a carbon sink in our study period and the average annual sum of the NEE was −1.30 tC ha^−1^ yr^−1^.

#### Representative diurnal patterns of carbon fluxes

**Figure 3:**
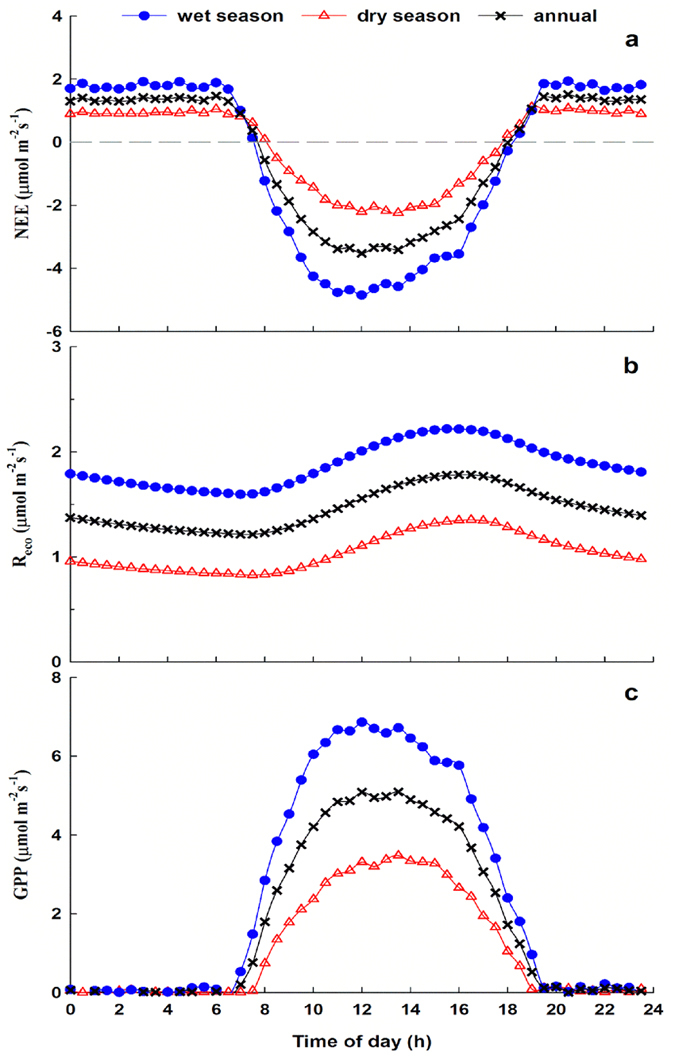
Seasonally binned (dry season, open triangles; wet season, blue circles) and yearly binned (black crosses) mean diurnal variations of (a) NEE, (b) R_eco_, and (c) GPP within the study plot.

Averaged over the year, the savanna ecosystem absorbed and fixed CO_2_ for ~9.5 hours per day (07:30–17:00). The savanna ecosystem becomes a carbon sink as the daily global radiation increases, and the average net maximum assimilations (i.e., NEE values) at 13:00 in the dry season, wet season, and annually were approximately 2.2, 4.6, and 3.4 μmol m^−2^ s^−1^, respectively. The rate of daytime CO_2_ fixation in the wet season is about twice that of the dry season (Fig. 3a). R_eco_ and GPP of the savanna ecosystem increased with increasing photosynthetically active radiation (PAR) and temperature after sunrise, reached their peaks at 16:00 (R_eco_) and 13:00 (GPP), and then decreased until sunrise the next day (Fig. 3b,c). The values of NEE, R_eco_, and GPP in the dry season are less than half those in the wet season (Fig. 3).

#### Monthly patterns of daytime NEE light response parameters

**Figure 4:**
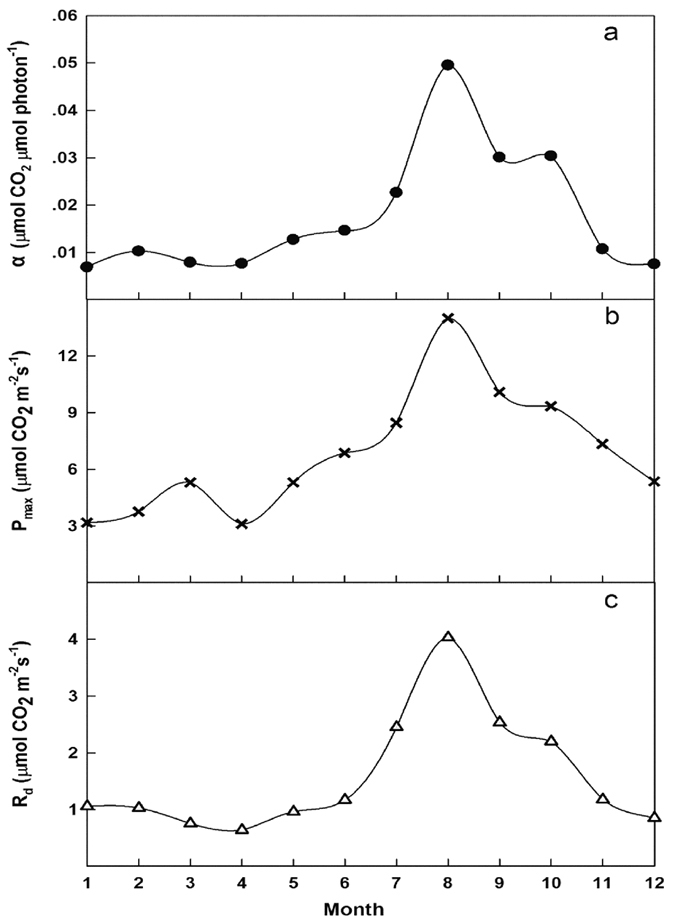
Average monthly variations in daytime NEE light response parameters from May 2013 to December 2015 in the savanna ecosystem in Southwest China. (**a**) Apparent quantum yield (α, μmol CO_2_ μmol photons^−1^); (**b**) maximum net photosynthetic rate (P_max_, μmol CO_2_ m^−2^ s^–1^); (**c**) dark respiration of the ecosystem (R_d_, μmol CO_2_ m^–2^ s^–1^).

Regarding monthly variations in daytime NEE light response parameters (Fig. 4), the apparent quantum yield (α) (Fig. 4a), maximum net photosynthetic rate (P_max_) (Fig. 4b) and dark respiration of the ecosystem (R_d_) (Fig. 4c) showed similar trends of monthly variation over the studied savanna ecosystem. The maximum and minimum α, P_max_, and R_d_ values were observed in August and April, respectively. In general, α, P_max_, and R_d_ values in the wet season (May-October) were higher than those during the dry season (November-April).

#### Seasonal daytime NEE responses to photosynthetically active radiation (PAR)

**Figure 5:**
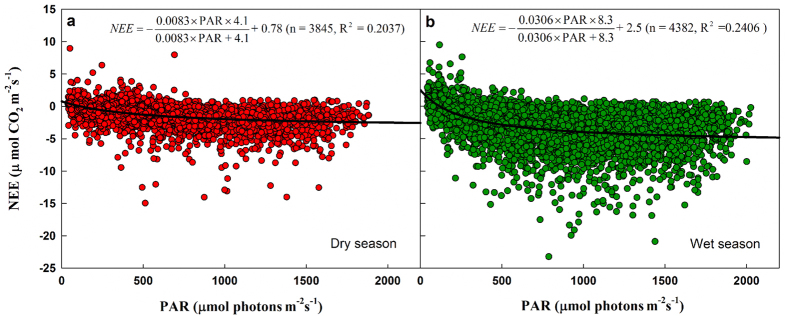
Response of dry and wet season daytime net ecosystem carbon exchange (NEE, μmol CO_2_ m^–2^ s^–1^) to photosynthetically active radiation (PAR). (**a**) Dry season (November–April); (**b**) wet season (May–October).

Carbon sequestration ability increased with increases in PAR irrespective of dry season or wet season (Fig. 5). However, the ecosystem showed higher (3.7 times) light transformation efficiency (photosynthetic capacity) in the wet season (0.0306) (Fig. 5b) than in the dry season (0.0083) (Fig. 5a), implying that most of the NEE (carbon sequestration amount) accumulated during the wet season (May-October).

#### Responses of NEE to temperature, monthly rainfall, RH, and VPD

**Figure 6:**
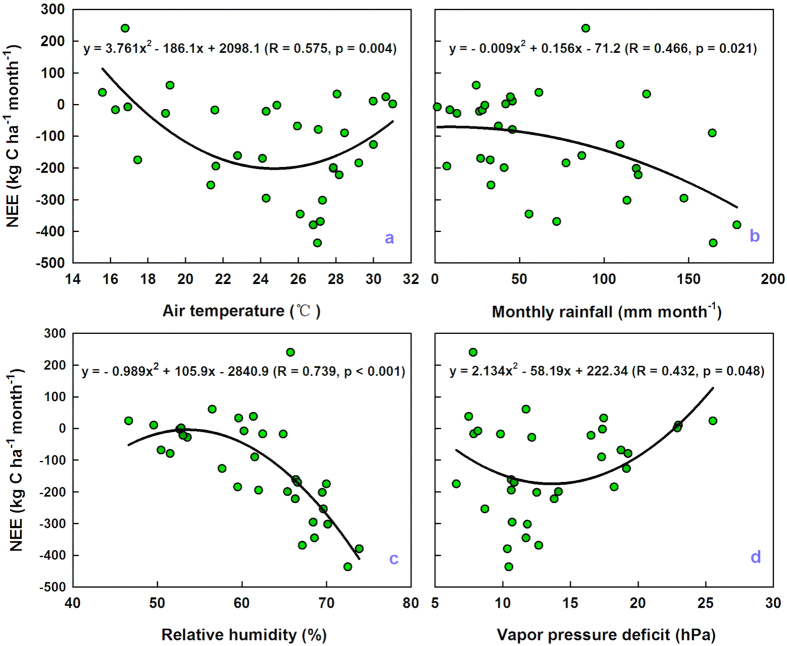
Responses of monthly total amount of net ecosystem carbon exchange (NEE, kg C ha^−1^ month^−1^) to (**a**) monthly mean air temperature (T_air_, °C), (**b**) total monthly rainfall (mm month^−1^), (**c**) monthly mean relative humidity (RH, %), and (**d**) monthly mean vapor pressure deficit (VPD, hPa) from May 2013 to December 2015 in the Yuanjiang savanna ecosystem, Southwest China. n = 32.

To explore the responses of net ecosystem carbon exchange (NEE, kg C ha^−1^ month^−1^) to environmental factors, a quadratic regression model was applied to make quantitative predictions of monthly NEE to monthly mean air temperature (T_air_), relative humidity (RH), vapor pressure deficit (VPD) and total monthly rainfall (Fig. 6). According to the regression results, carbon sink capacity increased with increasing T_air_, but decreased rapidly when T_air_ was higher than 24.7 °C (Fig. 6a); a similar trend was observed between NEE and VPD, in that carbon sequestration capacity decreased when VPD was higher than 13.7 hPa (Fig. 6d). Further, the carbon sink capacity of our study area decreased with decreasing RH and monthly rainfall (Fig. 6b,c).

## Discussion

### Annual carbon exchange

**Figure 7:**
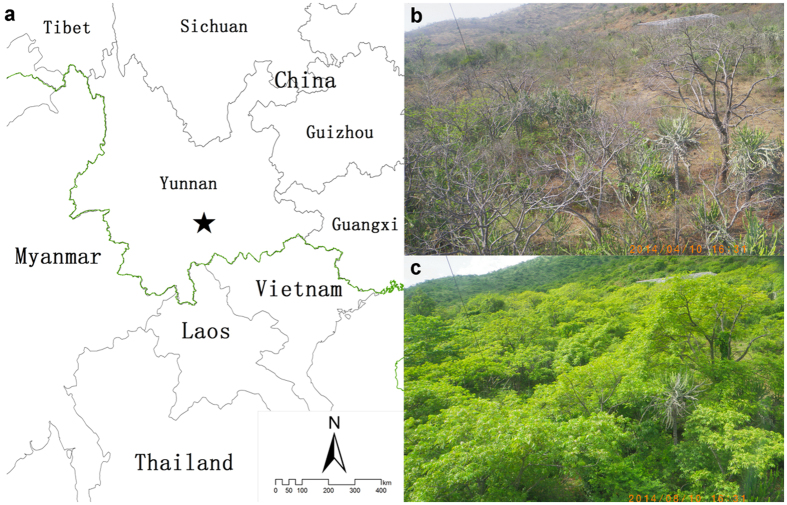
Geographical location and vegetation of the study site. (**a**) Location of the study site (black star); (**b**) savanna vegetation in the dry season and (**c**) in the wet season. The map (**a**) was generated using ArcGIS 9.3 software (ESRI Inc., Redlands, CA; http://resources.arcgis.com/en/home/) and the photographs (b & c) of savanna vegetation (canopy height ~8 m) were taken using a camera mounted at 13 m on the flux tower (13.9 m) at our study site.

Both the biometric method (BM) and the eddy covariance (EC) method were applied to quantify carbon exchange at the present study site (Fig. 7), although the two methods are different in terms of their spatial scales, temporal resolutions, the assumptions made by both techniques, as well as their advantages and flaws. EC is not only a less disturbing or non-destructive way to investigate carbon exchange, but also provides a dataset with high spatial resolution. This dataset covers time scales ranging from seconds to years^43^. In addition, EC can usually cover a larger spatial scale than BM and has better spatial representativeness^44^,^45^. However, EC assumes that the underlying surface should be horizontally homogenous^46^,^47^. This assumption is extremely difficult to satisfy, because forest ecosystems are comprised of heterogeneous canopy and terrain features. Therefore, as a conventional approach, it is necessary to apply BM simultaneously with EC at our study site, although there are some inherent flaws (including the extensive field work required, the indirect nature of the measurements, the method is more destructive, etc.). Furthermore, it is necessary to use the BM method to study ecosystem carbon exchange while tracking the contribution to NEP from each carbon pool in order to calculate forest carbon use efficiency.

**Figure 8:**
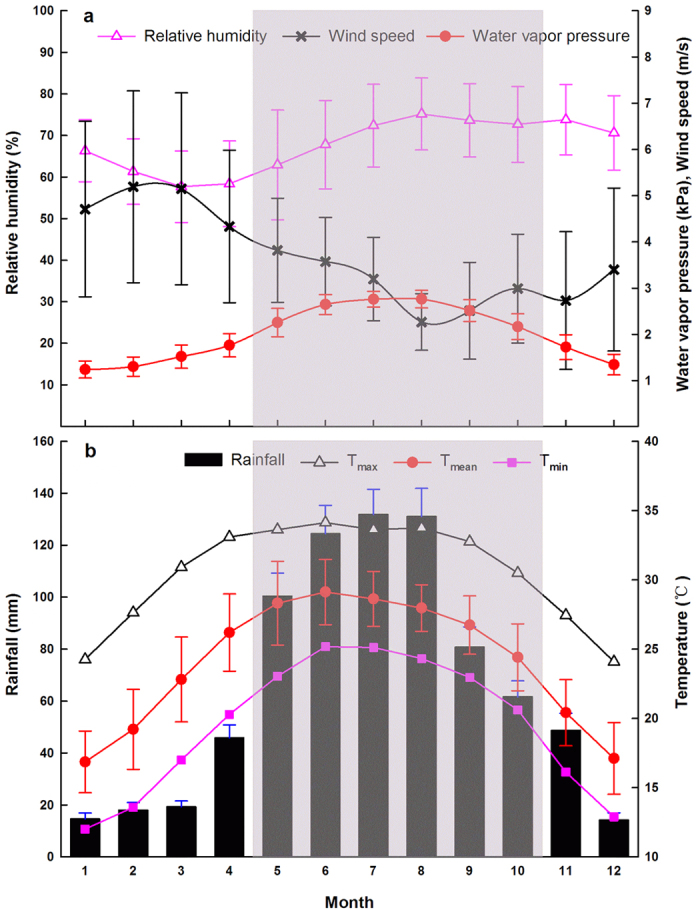
Seasonal pattern of long-term monthly means of meteorological data for the period 1980–2015 from Yuanjiang weather station, which is located ~20 km northwest of the study site. (**a**) Relative humidity (open triangles), water vapor pressure (red circles), and wind speed (black crosses); (**b**) monthly rainfall (black bars), monthly minimum air temperature (pink rectangles), monthly average air temperature (red circles) and monthly maximum air temperature (hollow triangles). Error bars represent 36 years of monthly standard deviations. Shaded area indicates the wet season (May–October) and the rest of the area is the dry season (November–April).

The results obtained using the BM (0.96 tC ha^−1^ yr^−1^) (Table 1) and EC (1.30 tC ha^−1^ yr^−1^) (Fig. 2) indicate that the site is an appreciable carbon sink, although under the control of high mean annual temperature (24.0 °C), high maximum mean monthly temperature (maximum MMT; 29.2 °C), but low mean annual rainfall (786.6 mm) (Fig. 8). There is a difference between the results of the biometric and eddy covariance methods that cannot be ignored. The discrepancy between them did not sufficiently indicate that the “lost” carbon amount of 0.34 tC ha^−1^ yr^−1^ (0.34 = 1.30–0.96) was fully stored as organic soil matter. The explanation for the discrepancy may be as follows. 1) The time periods covered by the EC observations (May 2013 to December 2015) differed from those of the biometric measurements (November 2013 to November 2015)^48^, which appears to be the most likely reason for the discrepancy^49^; 2) the allometric equations^50^ were not site-specific; 3) the flux footprint and the inventory plot were not exactly identical; 4) there is a time lag between tree growth derived from the BM and ecosystem photosynthesis determined from EC^44^; and 5) NEP measured by BM is usually lower than EC results under the conditions of well-developed turbulence^44^,^48^,^51^. In summary, temporal mismatch, the allometric equations, and the inventory are the three main reasons for the discrepancy between BM and EC results. Nevertheless, it is conceivable that the savanna ecosystem could be treated as a carbon sink regardless of which method is used. The EC result was more reasonable, although there are some uncertainties caused by lower turbulence on calm nights, advection, and possible cold air drainage of CO_2_^43^,^52^,^53^,^54^,^55^. Therefore, it is important to consider the plausibility of the eddy flux of net ecosystem carbon exchange at the study site.

Is it reasonable that the savanna ecosystem absorbed and fixed ~1.30 tC ha^−1^ in a year with strong seasonality ? Our answer is positive for the following reasons. 1) We followed strictly the ChinaFLUX procedures for QA/QC (quality assurance and quality control) and post-processing of the data to ensure reliable flux estimates^43^. Furthermore, an online procedure^53^, recommended by FLUXNET and maintained by the Max Planck Institute, was applied for gap filling and partitioning of the flux data with the widely used (particularly for forest systems) friction velocity threshold of 0.2^52^,^55^. 2) The biometric inventory result (Table 1), which was conducted within the footprint (Fig. 1) is consistent with the EC result, although there is some difference between the values. 3) The Ailaoshan subtropical evergreen broadleaf forest (24°32′N, 101°01′E, 2476 m a.s.l.) acts as a carbon sink of ~9 tC ha^−1^ yr^−1^ with little seasonality due to the lower mean annual temperature (MAT) and abundant mean annual rainfall (MAR)^44^. The amount of carbon sequestration at the present study site is just one-seventh of their result, and both sites are located in Yunnan province, Southwest China, in a high mountainous area (our study site is located in the valley, and the Ailaoshan study site is at the top of a mountain). 4) A comparison of carbon exchange (Table 2) shows us that the range of NEE in some global savanna ecosystems is from approximately 127.8 to −387.7 gC m^−2^ yr^−1^. Most savanna ecosystems act as carbon sinks and take up CO_2_ from the atmosphere by photosynthesis. The average NEE, GPP, and R_eco_ for these savanna ecosystems were −134.3 ± 158.3, 1012.6 ± 466.4, and 878.4 ± 378.7 gC m^−2^ yr^−1^, respectively, with an average annual rainfall of 716.3 ± 452.3 mm and a maximum MMT of 31.2 ± 3.2 °C. In an Australian *Acacia* woodland savanna^17^, an MAT and an MAR of 25.0 °C and 374.5 mm, respectively, were recorded during a research period of 2 years, and the net CO_2_ uptake amount reached 125 gC m^−2^ yr^−1^ with a GPP of 596.0 gC m^−2^ yr^−1^. Furthermore, the average GPP from 21 savanna sites in Australia was 687.97 ± 257.31 gC m^−2^ yr^−1^ 37. Thus, the averaged NEE, GPP, and R_eco_ values of −130, 684, and 554 gC m^−2^ yr^−1^, respectively, under conditions including a maximum MMT of 29.2 ± 0.58 °C and an MAR of 786.6 ± 153.2 mm at the present study site, are convincing and robust results.

### Seasonal variations in carbon exchange

We have shown that the savanna ecosystem in our study site acts as a carbon sink, that it absorbed approximately 1.30 tC ha^−1^ yr^−1^ from the atmosphere by means of photosynthesis, and that this result is reasonable and convincing. We next consider whether the seasonal variation in carbon fluxes is also reasonable. The 32-month (May 2013 to December 2015) averaged NEE of the wet season (−1.14 tC ha^−1^ yr^−1^) is ~7 times that of the dry season (−0.16 tC ha^−1^ yr^−1^) (Fig. 2). Interestingly, the dramatic seasonal variations in NEE, GEP, and R_eco_ values in Africa, Australia, and Brazil are also highly consistent with our results^9^,^15^,^21^,^23^,^25^,^34^,^56^. A study of a West African savanna reported a carbon source of 47.72 gC m^−2^ in the dry season but a carbon sink of −374.49 gC m^−2^ in the wet season in 2008^23^. Furthermore, in a semi-arid sparse savanna in Demokeya, Sudan, the daily amplitude of NEE in the wet season (−1.8 gC m^−2^ day^−1^) was 9 times that in the dry season (−0.2 gC m^−2^ day^−1^)^25^; the factor reached 20 in a tropical savanna in Australia^15^. Therefore, it is reasonable and not surprising that 87.7% of the NEE is absorbed in the wet season (−1.14 tC ha^−1^ yr^−1^) while the dry season is nearly carbon neutral in the savanna ecosystem at our study site.

The second question is why four-fifths of the NEE is taken up in the wet season. The reasons are as follows. The daytime NEE responses to photosynthetically active radiation (PAR) tell us that, although the dark respiration of the ecosystem (R_d_) increased during the wet season (May–October) (Fig. 4c), both light use efficiency (α) (Fig. 4a) and maximum net photosynthetic rate (P_max_) increased in the wet season (Fig. 4b). The wet- to dry-season ratio of α was 3.13 (0.0267 in the wet season and 0.0085 in the dry season), and P_max_ (3.11–13.98 μmol CO_2_ m^−2^ s^−1^) reached its peak (13.98 μmol CO_2_ m^−2^ s^−1^) in August. In addition, GEP and NEE increased rapidly with the coming of the wet season, and peak GEP and NEE were −6.09 and −3.03 gC m^−2^ d^−1^ on 30 August (Fig. 2), respectively. Furthermore, the net assimilation of carbon increased dramatically in the wet season compared with the dry season (Fig. 5), and previous studies have also shown higher photosynthesis rates in the wet season than in the dry season^57^. Therefore, the fact that most of the annual NEE accumulated during the wet season in our research area is reasonable and convincing.

### Climate change and carbon exchange

**Figure 9:**
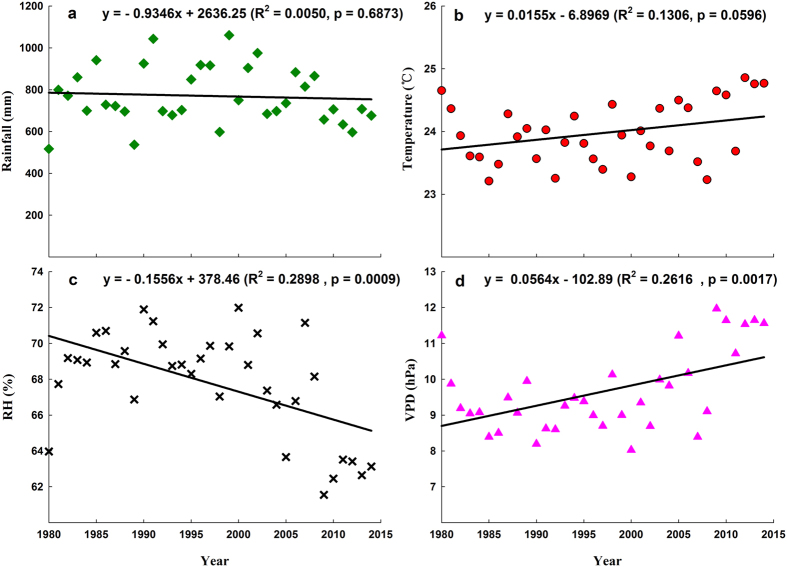
Temporal trends in environmental factors between 1980 and 2015 derived from a weather station located ~20 km northwest of the study site. (**a**) Annual rainfall (mm); (**b**) mean annual temperature (°C); (**c**) relative humidity (RH, %); (**d**) vapor pressure deficit (VPD, hPa).

The savanna ecosystem at our study site acts as a carbon sink of 1.30 tC ha^−1^ yr^−1^ in the global carbon cycle, with approximately 88% of this carbon being absorbed during the wet season (May–October), while it is nearly carbon neutral in the dry season (Fig. 2). Carbon sink capacity decreases with increasing T_air_ and VPD and decreasing rainfall and RH (Fig. 6). Therefore, it is important to consider the impacts of future climate changes on carbon exchange in such a savanna ecosystem, as its severe environment may be highly sensitive to changes in rainfall and temperature^5^,^30^, and many previous studies have revealed that water and temperature have important impacts on savanna ecosystem carbon exchange^5^,^8^,^13^,^29^,^34^,^37^,^58^,^59^,^60^. Observations show that, over the past 36 years (1980–2015), the climate in the present study site has become hotter and drier with increasing T_air_ and VPD, while annual rainfall and RH show decreasing trends (Fig. 9). In addition, there was a significant contradiction between water and heat, with an increasing shortage of rainfall (Fig. 9) and abundance of net radiation^42^,^61^. Therefore, the carbon sequestration ability of the savanna ecosystem will decrease (Fig. 6) under decreasing rainfall and increasing temperature (Fig. 9). We should, therefore, pay close attention to protecting similar savanna ecosystems and specific research should assess the influence of climate change on carbon and water exchanges.

## Conclusions and Prospects

The biometric-based method (BM) and eddy covariance technique (EC) were used to determine carbon exchange over a savanna ecosystem in Southwest China. Our results and the discussion above lead to the following preliminary conclusions.

First, the carbon use efficiency (CUE = NPP/GPP)^62^,^63^,^64^ was 0.60 (4.11/6.84), slightly higher than the mean CUE of all forests (0.53), which varies from 0.23 to 0.83^65^. Second, the largest daily net carbon release (22 January) and the maximum carbon sink (30 August) were 1.57 and −3.03 gC m^−2^ d^−1^ (equivalent to 1.51 and −2.92 μmol m^−2^ s^−1^), respectively. Third, the carbon exchange varies dramatically between the dry season (when the savanna is nearly carbon-neutral or a small carbon sink of 0.16 tC ha^−1^ yr^−1^) and the wet season (when the savanna is an appreciable carbon sink of 1.14 tC ha^−1^ yr^−1^) based on the post-QA/QC EC results. Fourth, savanna ecosystems act as an appreciable carbon sink in the global carbon cycle according to both BM (0.96 tC ha^−1^ yr^−1^) and EC (1.30 tC ha^−1^ yr^−1^) results. Fifth, GPP, R_eco_, and NEE were 6.84, 5.54, and −1.30 tC ha^−1^ yr^−1^, respectively, at our study site during May 2013 to December 2015. At a global scale (Table 2), the mean GPP, R_eco_, and NEE were 10.13 ± 4.66, 8.78 ± 3.79, and −1.34 ± 1.58 tC ha^−1^ yr^−1^, respectively. Consequently, the carbon sink strength of this savanna was close to the mean carbon sink ability of savannas globally. Note that the carbon sequestration capacity (i.e., the amount of CO_2_ that the savanna ecosystem can take up) will decrease in the future under ongoing climate change (Fig. 6) as the climate here becomes hotter and drier than in past decades (Fig. 9). Therefore, it is critical that corresponding policies or management practices should protect similar savanna ecosystems that are subjected to decreasing rainfall amounts and rising temperatures. Further studies, which in turn help protect this area, should be conducted to understand the extent of the influence of climate change and the mechanisms responsible for this influence on energy, carbon, and water fluxes in the region.

## Materials and Methods

### Experimental site description

#### Site description

The geographical location of our research site (23°28′25.93″N, 102°10′38.76″E; 553 m a.s.l.) is in Yuanjiang Nature Reserve (YNR) in Yunnan province, Southwest China (Fig. 7a). The slope of the study plot terrain is ~15° and the soil is classified as torrid red earth (dry red soil).

Hot-dry winds dominate the climate due to the Foehn effect and the enclosed nature of the topography^41^,^66^,^67^, so the climate here is dry and hot with a high average annual temperature and low average annual rainfall, and there is considerable savanna vegetation spread throughout the area. The phenology shifts dramatically because of the distinct changes between the dry and wet seasons (Fig. 7b,c). The period of leaf-fall is mainly between the end of the rainy season and the middle of the dry season, and most leaves are shed before the start of the driest month, even though the trees are dry-season deciduous species^68^ (Fig. 7b). Vegetation growth is strongest in the middle of the wet season (August) (Fig. 7c).

A permanent savanna ecological research plot (1 ha) associated with the Yuanjiang Savanna Ecosystem Research Station (YSERS) of the Xishuangbanna Tropical Botanical Garden of the Chinese Academy of Sciences, was established on a west-facing slope in YNR in 2011, as described in previous studies^41^,^67^, and the YSERS carried out an investigation of the vegetation in 2012. The savanna vegetation (canopy height of ~8 m) here consists mainly of small trees, shrubs, and herbs. In this community, the dominant trees are *Lannea coromandelica, Polyalthia suberosa, Diospyros yunnanensis* and similar species. The dominant shrubs are *Vitex negundo* f. *laxipaniculata, Campylotropis delavayi, Woodfordia fruticosa, Euphorbia royleana, Jasminum nudiflorum, Tarenna depauperata*, etc. The dominant herbaceous species are *Heteropogon contortus* and *Bothriochloa pertusa*^41^,^67^,^68^,^69^. As an adaptation to the region’s high temperature and low rainfall, the leaves of these species are relatively small, with thick cuticle and smooth or waxy leaf surfaces.

#### Long-term meteorological conditions and regional climate patterns

Thirty-six years (1980–2015) of meteorological records (Fig. 8) from a weather station located ~20 km northwest of the study site give the monthly variations in relative humidity (RH), water vapor pressure (e), wind speed (WS), rainfall, minimum air temperature (T_min_), mean air temperature (T_mean_), and maximum air temperature (T_max_). Overall, the results show that the wet season RH, e, rainfall, T_min_, T_mean_, and T_max_ are larger than the dry season values, but WS is lower in the wet season.

According to the long-term results (Fig. 8b), the mean annual temperature (MAT) is 24.0 ± 0.5 °C, and the monthly average temperatures of the coldest month (January) and the hottest month (June) are 16.9 ± 2.2 °C and 29.2 ± 2.4 °C, respectively. The climate is strongly seasonal; in the wet season (May–October), the climate is dominated by the tropical southern monsoon from the Indian Ocean, which delivers most of the annual rainfall (786.6 ± 153.2 mm). The ratio of wet season rainfall to annual rainfall can reach 81.0%, whereas in the dry season (November–April), the total rainfall is less than 150 mm. There are more than 100 days with temperatures above 35 °C in the YSERS records for 2012–2013^69^. The yearly total number of sunshine hours is 2261.7^61^, the annual average pan evaporation is 2750 mm^41^, and the aridity index (AI) is ~0.29. These values indicate that the study area belongs to the semi-arid class according to the definition of semi-arid regions (AI = 0.2–0.5)^70^.

#### Climate change trends in our study area

The results of 36 years (1980–2015) of observations on temporal trends in rainfall, temperature, RH, and VPD (Fig. 9) showed that the observed declining trend in rainfall (p = 0.6873) and the observed increasing trend in temperature (p = 0.0596) were not statistically significant (Fig. 9a,b). However, both the RH (p = 0.0009) and VPD (p = 0.0017) increased significantly (Fig. 9c,d). Therefore, the climate here is becoming drier and hotter than it previously was, and the opposite trends seen in water supply and heat are becoming exacerbated^42^.

### Biometric and eddy covariance method for estimate of carbon exchange

#### Biometric-based NEP estimation

The biometric method is a conventional way of estimating NEP all over the world^49^,^51^,^71^,^72^,^73^, and the expression^74^,^75^,^76^ for NEP as estimated by the biometric method is


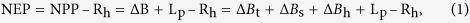


where NEP is generally defined as the net ecosystem production that represents the balance between GPP and ecosystem respiration (R_eco_); NPP is net primary production, with R_h_ the heterotrophic respiration of the ecosystem; ΔB is the biomass increment; L_p_ is above-ground litterfall production; and ΔB_t_, ΔB_s_, and ΔB_h_ are the biomass increments of trees, shrubs, and herbs, respectively.

To estimate the biomass production, we inventoried 1 ha of vegetation within the footprint of the eddy flux tower in November 2013. All trees with diameter at breast height (DBH) > 2 cm were identified, tagged, measured (in terms of their height and DBH), and mapped. Standard allometric equations for karst vegetation^50^ were used to calculate tree biomass from DBH and height, because site-specific allometric equations were not available. Carbon density was derived from the biomass by multiplying by a factor of 0.5^77^,^78^. In November of 2014 and 2015, we re-measured tree DBH and the heights of trees tagged and measured in 2013 to estimate components of the biomass carbon budget including DBH increment, tree height, recruitment, growth, mortality, and coarse woody debris. Litterfall was captured by 20 litterfall traps (1 m × 1 m) that were randomly located in the 1 ha permanent ecological research plot. The litterfall was collected on the last day of each month and then sorted into leaves, branches, flowers, and fruits. Each component was dried to a constant weight at 65 °C, then weighed and recorded. For the calculation of ΔB_s_ and ΔB_h_, the harvest method was used to estimate the above-ground and below-ground biomass of shrubs (2 m × 2 m with 3 sets and 4 repeats) and herbs (1 m × 1 m with 5 sets and 3 repeats) near the 1 ha permanent plot.

Root removal by trenching was used to measure R_h_^76^ within the footprint of the flux tower. The volume of root trenching was 100 cm × 100 cm × 40 cm and the volume was wrapped with wire mesh (0.149 mm × 0.149 mm) to prevent the growth of new roots. Two treatments (CK and root trenching) were applied with six replicates in August 2014. Open-top static chambers (60 cm × 32 cm × 30 cm) together with a gas chromatograph (7890D GC, Agilent Co. Produced, USA) were used to measure R_h_. Sampling was performed (~4 months after root trenching) twice a month from Nov 2014 to Dec 2015 (usually the 15^th^ and the last day of each month) during 09:00 and 11:00 each day.

#### Eddy covariance-based NEE estimations

##### Eddy covariance and meteorological measurement system

Eddy covariance provides a direct and continuous measure of matter and energy fluxes between an ecosystem and the atmosphere^79^,^80^, and has been applied across the globe to different cover types including forests, farmlands, grasslands, wetlands, tundras, deserts, and aquatic ecosystems to measure energy, carbon, and water exchanges^81^. The EC and meteorological instruments were mounted and oriented in the prevailing wind direction at an angle of 135° from north at a height of 13.9 m on a flux tower that was established near the 1 ha permanent plot in April 2013. The eddy covariance system characteristics and parameters used in this paper are as follows. 1) The EC system consisted of a triaxial sonic anemometer (CSAT3, Campbell Scientific Inc., USA) and a high-frequency open-path CO_2_/H_2_O infrared gas analyzer (Li-7500, Li-Cor Inc., USA) installed at a height of 13.9 m. 2) Measurements of wind speed (A100R, Denbighshire, UK) and direction (W200P, Denbighshire, UK) were made at two heights, and a photosynthetically active radiation (LQS70–10, APOGEE, USA) profile measurement system was also deployed. The sampling frequencies of flux data and meteorological data were 10 Hz and 0.5 Hz, respectively. Control systems were used for the simultaneous acquisition of flux data (model CR1000, Campbell Scientific Inc., Logan, UT, USA) and meteorological data (model CR5000, Campbell Scientific Inc.). All data were collected continuously beginning in May 2013.

##### NEE Calculation

The NEE between the forest ecosystem and atmosphere is the sum of the turbulent eddy flux and the storage flux^79^,^82^,^83^ as equation (2):


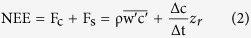


where F_c_ is the turbulent eddy flux transported between the EC measurement plane above the forest and the atmosphere, F_s_ indicates the storage flux under the plane of the eddy covariance system (13.9 m in this study), ρ is air density, w is the vertical wind velocity, c represents CO_2_ concentration measured by an infrared gas analyzer, the primes denote fluctuations in the target scalar (CO_2_ concentration in this case) from the average, and the overbar signifies a time average (30 min in this case). Δc is the variation in CO_2_ concentration over a 30 min period at height z_r_, Δt is the time interval (1800 s in this case) and z_r_ is the height of the plane of the eddy covariance system above the ground (13.9 m in this case). Generally, negative NEE values indicate that the ecosystem fixes CO_2_ from the atmosphere by photosynthesis and acts as a carbon sink. Thus, NEE is generally equal to −NEP.

##### Data processing and carbon flux calculation

Quality assessment and control (QA/QC) are necessary to ensure the reliable processing of flux data before calculating energy, carbon, and water fluxes to account for environmental and meteorological limitations (topography, rain, advection, and low turbulence issues). ChinaFLUX has developed a series of standard methodologies to assess the EC system and to control the quality of flux data. For details of data QA/QC and post-processing procedures used in the present study, see ref. 43. Here, we briefly introduce the data processing flow. 1) Three-dimensional coordinate rotation was applied to remove the effects on airflow of instrument tilt or irregularities in the terrain^84^,^85^,^86^; 2) In WPL calibration, flux data were corrected for air density variations arising from the transfer of heat and water vapor^87^; 3) data recorded in rainy periods were discarded^43^; 4) storage flux (F_s_) was calculated^79^,^82^,^88^; 5) outliers were identified and eliminated^53^, and absolute NEE values > 50 μmol m^−2^ s^−1^ (i.e., NEE values larger than 50 or less than −50 μmol m^−2^ s^−1^) were rejected^44^; 6) negative nighttime data were rejected; 7) data with friction velocities (u*) < 0.2 were filtered^52^,^55^; and 8) gap filling and partitioning were applied to the flux data^52^,^53^,^54^ using an online procedure that is recommended by FLUXNET and used as standard by EUROFLUX and maintained by the Max Planck Institute (http://www.bgc-jena.mpg.de/~MDIwork/eddyproc/index.php).

## Additional Information

**How to cite this article:** Fei, X. *et al*. Eddy covariance and biometric measurements show that a savanna ecosystem in Southwest China is a carbon sink. *Sci. Rep.* **7**, 41025; doi: 10.1038/srep41025 (2017).

**Publisher's note:** Springer Nature remains neutral with regard to jurisdictional claims in published maps and institutional affiliations.

## Supplementary Material

Supplementary Figures

## Figures and Tables

**Table 1:** Carbon budget (tC ha^−1^ yr^−1^) and carbon use efficiency (CUE) between November 2014 and November 2015 in the Yuanjiang savanna ecosystem in Southwest China (a: GPP is the mean value derived from the eddy flux during the study period).

Parameter	Trees	Shrubs	Herbs	Total
Carbon storage 2014 (tC ha^−1^ yr^−1^)	26.35	2.65	2.77	
Carbon storage 2015 (tC ha^−1^ yr^−1^)	27.43	3.21	3.10	
Biomass increment (tC ha^−1^ yr^−1^)	1.08	0.56	0.33	
ΔB (tC ha^−1^ yr^−1^)				1.97
L_p_ (tC ha^−1^ yr^−1^)				2.14
R_h_ (tC ha^−1^ yr^−1^)				3.16
NEP (tC ha^−1^ yr^−1^)				0.96
NPP (tC ha^−1^ yr^−1^)				4.11
GPP (tC ha^−1^ yr^−1^)^a^				6.84
CUE (dimensionless)				0.60

**Table 2:** Comparison of carbon exchange (NEE, GPP, R_eco_, gC m^−2^ yr^−1^) in savanna ecosystems worldwide.

Country/Area	Location	Latitude & longitude	MAR	T_air_	Vegetation	NEE	GPP	R_eco_	References
Sudan	Sumbrugu Aguusi	10°50′45.6″N, 0°55′1.2″W	375.0	23.3/34.7	grassland savanna	127.8 ± 7.2	874.0 ± 17.8	1001.8 ± 19	34
Sudan	Kayoro Dakorenia	10°55′4.8″N, 01°19′15.6″W	—	22.0/34.9	fallow and cropland	108.0 ± 5.5	781.3 ± 15.8	889.3 ± 16.5	34
South Africa	Kruger Park	—	582.4 ± 170.9	17.5/26.0	semi-arid savanna	25.2 ± 133.3	—	—	13
Australia	Virginia Park	19°53′00″S, 146°33′14″E	571.0	17.1/30.1	semi-arid savanna	21	576	597	33
Spain	El Llano de los Juanes	36°55′41.7″N, 02°45′1.7″W	227.0	MAT: 12.0	Mediterranean shrubland	−2 ± 23	—	—	24
Southern Africa	Ca. 20 km east of Maun, Botswana	Maun, Botswana (23°33′E, 19°54′S)	464.0	14.9/30.3	woodland savanna	−12.0	386	374	9
China	Yanchi Research Station,	37°42.51′N, 107°13.62′E	305.0	MAT: 8.1	semi-arid shrub	−77.0	456	379	58
United States	Tonzi Ranch, California	38°25′48″N, 120°57′00″W	562.1 ± 193.3	8.1/26.9	oak and grass savanna	−98 ± 51	1070 ± 193	972 ± 186	26
Australia	Pine Hill cattle station	22°16′48″S, 133°15′00″E	374.5	MAT: 25.0	woodland savanna	−125	596	471	17
China	Yuanjiang Savanna Ecological Station	23°28′26″N, 102°10′39″E	786.6	16.9/29.2	semi-arid savanna	−130	684	554	This study
Australia	Howard Springs	12°30′24″S, 131°5′24″E	1487.0	23.2/31.9	mesic savanna	−155	1740	1585	33
Sudan	Northwestern Benin	09°44′24″N, 01°36′00″E	1190.0	MAT: 24.0	cultivated savanna	−232 ± 27	1593 ± 52.3	1360.9 ± 28.7	23
Australia	central Australia	22°18′00″S, 133°12′00″E	318.3	MDT: 8.0/34.4	*Acacia* savanna	−257.8	—	—	22
West Africa	Dahra field site	15°24′00″N, 15°24′48″W	524.4	25.0/32.0	shrub and tree savanna	−270.8 ± 47	1043 ± 137	772 ± 96	12
Brazil	Reserva Ecológica do IBGE	15°56′S, 47°51′W	1017.0	19.0/26.0	trees and shrubs	−288	1272	984	21
Southern Sudan	Bontioli	10°51′56″N, 03°4′22″W	852.0	24.8/32	trees and shrub savanna	−304	—	—	10
Australia	Howard Springs	12°29.712′S, 131°09.003′E	1824.0	20.0/33.6	open-forest savanna	−360.0 ± 38	1380 ± 38	1020 ± 11	19
Sudan	Nazinga Park	11°09′7.20″N, 1°35′9.6″W	—	22.6/34.5	nature reserve savanna	−387.3 ± 23	1725.1 ± 33	1337.8 ± 23	34

MAR is mean annual rainfall (mm). In the column labeled T_air_ (°C), MDT is mean daily temperature, MAT is mean annual temperature, and the others are min.MMT/max.MMT (minimum mean monthly temperature to maximum mean monthly temperature). The sites are listed in descending order of NEE (a positive value means the ecosystem is a carbon source, and a negative value indicates a carbon sink that takes up CO_2_ from the atmosphere). The values of NEE, GPP, and R_eco_ listed in this table are shown as the mean value ± the standard deviation (sd) over the study period.
