# Supplementary material for: Eddy covariance and biometric measurements show that a savanna ecosystem in Southwest China is a carbon sink
Source: Sci Rep. 2017 Feb 1;7:41025. doi: 10.1038/srep41025 (PMC5286525; doi:10.1038/srep41025)
Supplement: Supplementary Figures [file srep41025-s1.doc]

# Eddy covariance and biometric measurements show that a savanna ecosystem in Southwest China is a carbon sink

Xuehai Fei^1, 2^, Yanqiang Jin^1, 2^, Yiping Zhang^1,*^, Liqing Sha^1^, Yuntong Liu^1^, Qinghai Song^1^, Wenjun Zhou^1^, Naishen Liang^4^, Guirui Yu^5^, Leiming Zhang^5^, Ruiwu Zhou^1, 2^, Jing Li^1, 2^, Shubin Zhang^1, 2, 3^ & Peiguang Li^1, 2, 3^

1. Key Laboratory of Tropical Forest Ecology, Xishuangbanna Tropical Botanical Garden, Chinese Academy of Sciences, Mengla, Yunnan 666303, China.
2. University of Chinese Academy of Sciences, Beijing 100039, China。
3. Yuanjiang Savanna Ecosystem Research Station, Xishuangbanna Tropical Botanical Garden, Chinese Academy of Sciences, Yuanjiang, Yunnan 653300, China.
4. Global Carbon Cycle Research Section, Center for Global Environmental Research, National Institute for Environmental Studies, Tsukuba, 305-8506, Japan.
5. Synthesis Research Center of Chinese Ecosystem Research Network, Key Laboratory of Ecosystem Network Observation and Modeling, Institute of Geographic Sciences and Natural Resources Research, Chinese Academy of Sciences, Beijing 100101, China

*Corresponding author: Yi-Ping Zhang

Email: [yipingzh@xtbg.ac.cn](mailto:yipingzh@xtbg.ac.cn)

Tel: +86-871-65160904

Fax: +86-871-65160916

Corresponding address: 88 Xuefu Road, Kunming, Yunnan, P.R. China

Postal code: 650223

# Fig.S1


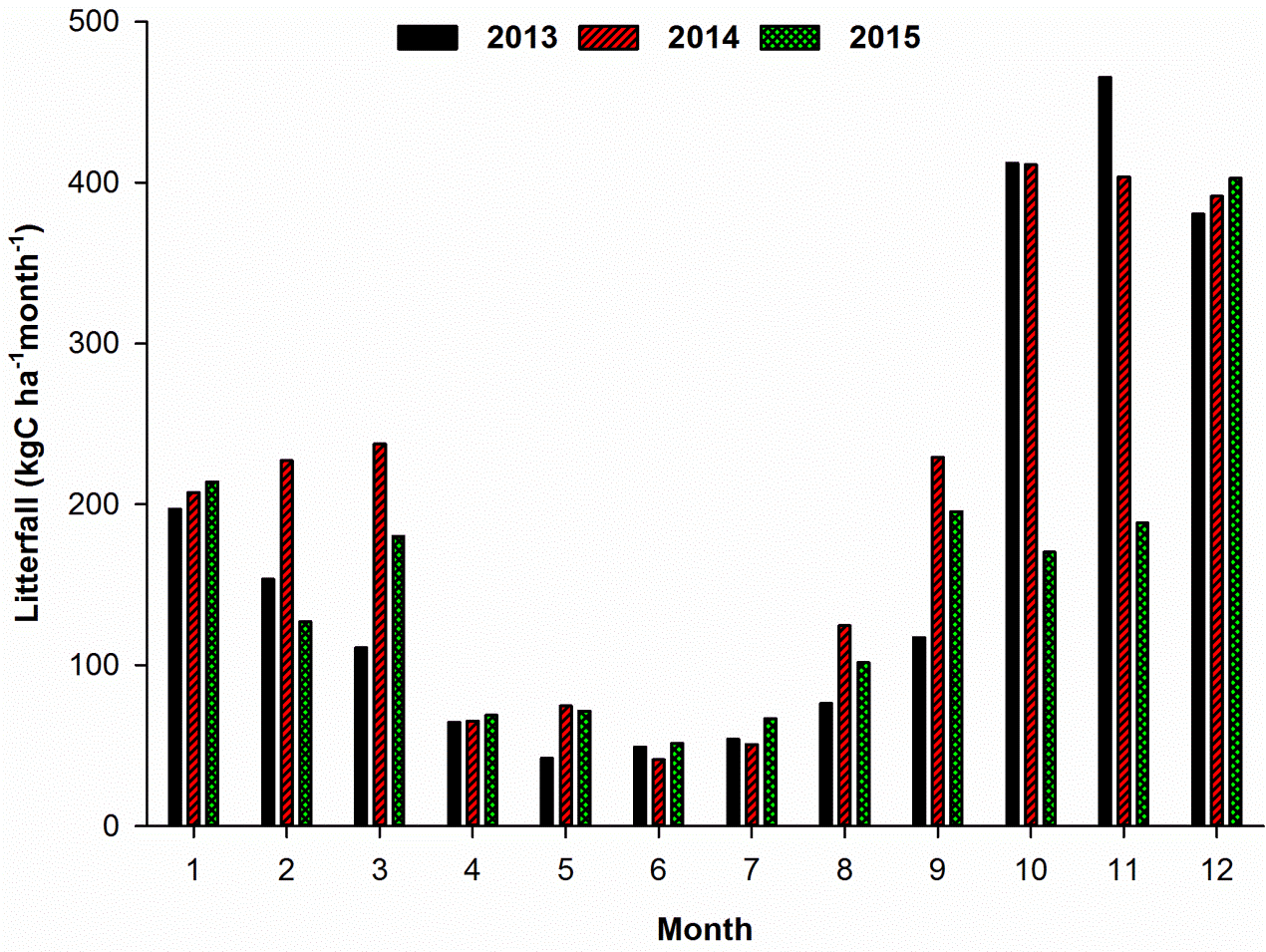


Fig.S1. Monthly and inter-year variation of litterfall from Jan 2013 to Dec 2015 at the study site. Strong seasonal (Wet season: May to October; Dry season: November to April) fluctuations in litterfall were observed. Increased rainfall in October and November 2015 resulted in much delayed litterfall compared to the other two years.

# Landscape and vegetation at the study site


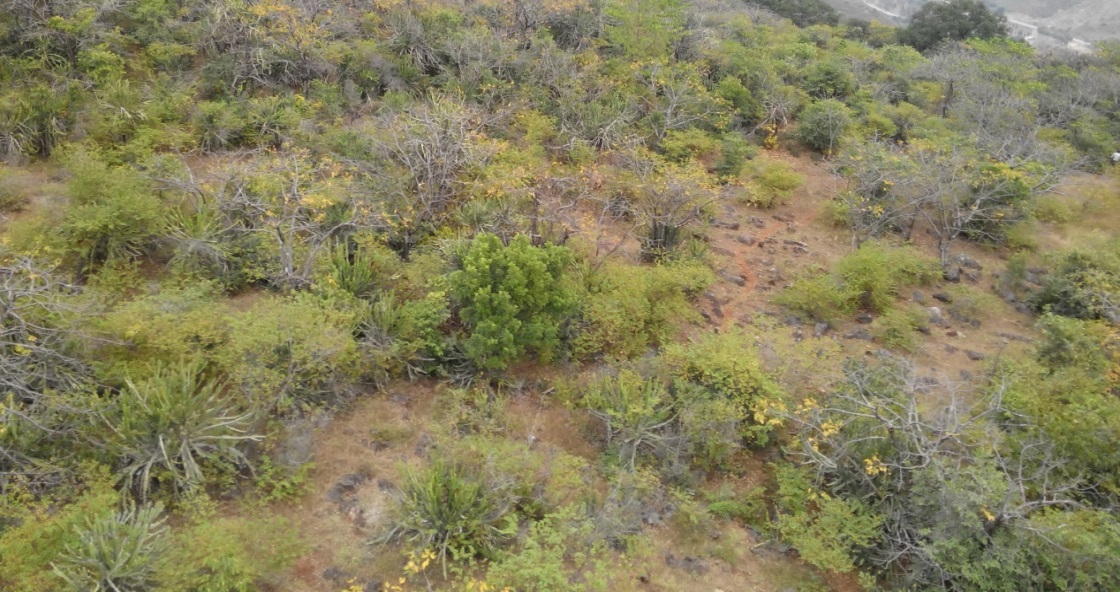


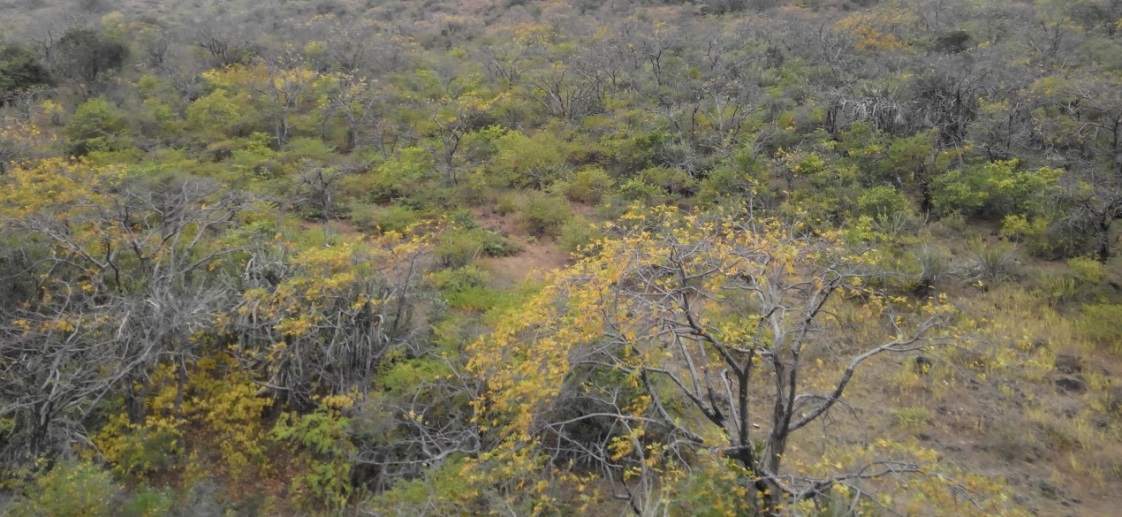


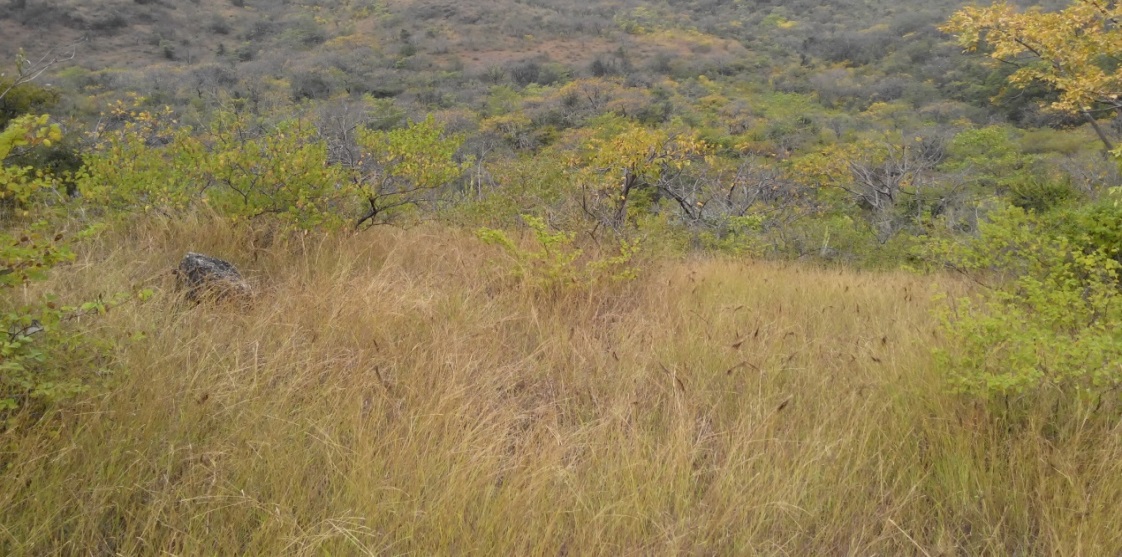


Photo group 1. The vegetation in the Yuanjiang semi-arid savanna ecosystem at the study site. The savanna vegetation (with canopy height of ~8 m) consists mainly of low trees, shrubs, and herbs. In this community, the dominant tree species are *Lannea coromandelica*, *Polyalthia suberosa*, and *Diospyros yunnanensis*. The dominant shrub species are *Vitex* *negundo* f*.* *laxipaniculata*, *Campylotropis delavayi*, *Woodfordia fruticosa*, *Euphorbia royleana*, *Jasminum* *nudiflorum*, and *Tarenna depauperata* etc. The dominant herbaceous species are *Heteropogon contortus* and *Bothriochloa pertusa*. To adapt to the high temperature and low rainfall in this region, the leaves of the vegetation are relatively small, with a thicker cuticle and smooth or waxy leaf surface.

# The eddy covariance and micrometeorological observation system at the study site


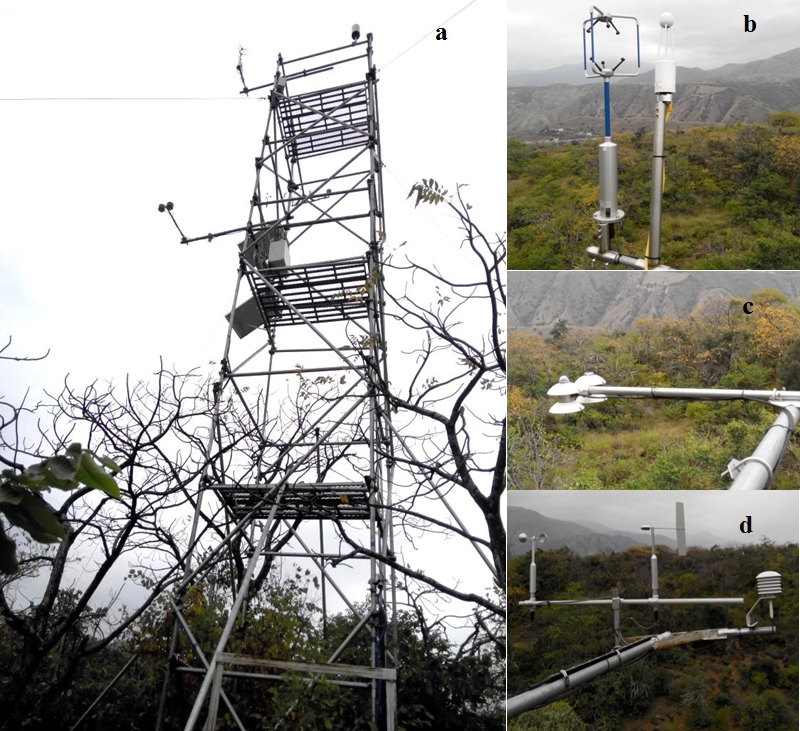


Photo group 2. The eddy covariance and micrometeorological observation system in the Yuanjiang semi-arid savanna ecosystem. (a) Flux tower; (b) eddy covariance system (13.9m); (c) solar radiation sensor; (d) profile system for microclimatic factor (wind direction, wind speed, air temperature, and humidity).
